# Development of Anti-Inflammatory Agents Utilizing DC-SIGN Mediated IL-10 Secretion in Autoimmune and Immune-Mediated Disorders: Bridging Veterinary and Human Health

**DOI:** 10.3390/ijms26052329

**Published:** 2025-03-05

**Authors:** Hayeon Baek, Seung-Woo Yang, Seulki Kim, Yunseok Lee, Hwi Park, Min Park, Byung-Ju Jeon, Hanwool Park, Han-Sung Hwang, Joon-Young Kim, Jung-Hyun Kim, Young-Sun Kang

**Affiliations:** 1 Department of KONKUK-KIST Biomedical Science & Technology, Konkuk University, 1 Hwayang-dong, Gwangjin-gu, Seoul 143-701, Republic of Korea; baekhy12@konkuk.ac.kr (H.B.); parkmin777@gmail.com (M.P.); 2 Sanford Consortium for Regenerative Medicine, School of Medicine, University of California, San Diego, CA 92037, USA; mdmichaelyang@gmail.com; 3 Division of Maternal and Fetal Medicine, Department of Obstetrics and Gynecology, Research Institute of Medical Science, School of Medicine, Konkuk University, 120 Neungdong-ro, Gwangjin-gu, Seoul 05029, Republic of Korea; hwanghs@kuh.ac.kr; 4 Department of Veterinary Pharmacology and Toxicology, Veterinary Science Research Institute, College of Veterinary Medicine, Konkuk University, 120 Neungdong-ro, Gwangjin-gu, Seoul 05029, Republic of Korea; youxoxo95@konkuk.ac.kr (S.K.); kgultong@konkuk.ac.kr (Y.L.); 5 Department of Veterinary Ophthalmology, College of Veterinary Medicine, Konkuk University, 120 Neungdong-ro, Gwangjin-gu, Seoul 05029, Republic of Korea; ehrdlfdndb@naver.com (H.P.); neru0512@naver.com (B.-J.J.); phwool123@naver.com (H.P.); canvet@hanafos.com (J.-Y.K.); 6 Department of Veterinary Internal Medicine, College of Veterinary Medicine, Konkuk University, 120 Neungdong-ro, Gwangjin-gu, Seoul 05029, Republic of Korea; junghyun@konkuk.ac.kr

**Keywords:** DC-SIGN, IL-10, anti-inflammatory effects, veterinary autoimmune diseases, veterinary immune-mediated diseases, DC-SIGN ligands, DC-SIGN homologues in animals, comparative medicine, translational research

## Abstract

DC-SIGN (dendritic cell-specific intercellular adhesion molecule-3-grabbing non-integrin) is a C-type lectin receptor expressed on dendritic cells and M2 macrophages, playing a key role in immune regulation and pathogen recognition. Its ability to mediate anti-inflammatory effects by interacting with specific ligands triggers pathways that suppress pro-inflammatory responses and promote tissue repair, making it a potential therapeutic target for inflammatory and autoimmune diseases. DC-SIGN homologs in various animal species share structural similarities and perform comparable immune functions, offering valuable insights into its broader application across species. By recognizing carbohydrate ligands on pathogens, DC-SIGN facilitates immune modulation, which can be harnessed for developing therapies aimed at controlling inflammation. In veterinary medicine, autoimmune and inflammatory diseases, such as rheumatoid arthritis and inflammatory bowel disease, represent significant challenges, and the anti-inflammatory properties of DC-SIGN could provide new therapeutic options to improve disease management and enhance animal health. Future investigations should focus on the structural and functional analysis of DC-SIGN homologs in various species, as well as the development of preclinical models to translate these findings into clinical interventions bridging veterinary and human health.

## 1. Introduction

Immune tolerance is crucial for maintaining the health and well-being of companion animals, serving as a protective mechanism that prevents the immune system from attacking the body’s own tissues while still allowing it to respond effectively to external pathogens [1]. When these immune tolerance mechanisms fail, the consequences can be severe, and autoimmune diseases arise as the immune system mistakenly targets self-antigens, causing tissue damage and chronic inflammation [2]. Therefore, immune tolerance is a critical process that protects companion animals from autoimmune diseases and chronic inflammation.

T cells, B cells, regulatory T cells (Tregs), macrophages, and dendritic cells (DCs) are central to maintaining immune tolerance and preventing autoimmune attacks. Tregs play a critical role by suppressing overactive immune responses, including self-reactive T cells, to protect the body from targeting its own tissues [3]. Macrophages contribute by adopting anti-inflammatory roles, particularly through the M2 phenotype, which supports tissue repair and minimizes inflammation [2,4]. DCs, as professional antigen-presenting cells, are pivotal in processing and presenting antigens to T cells, fostering Treg development and preventing the activation of self-reactive T cells [4]. Together, these cells maintain the delicate balance between immune activation and regulation, ensuring normal immune function and protecting against autoimmune diseases [2]. Dysfunction in macrophages or DCs can disrupt this balance, leading to the breakdown of immune tolerance and the development of immune-mediated conditions such as Canine Rheumatoid Arthritis, Pemphigus Complex, autoimmune hemolytic anemia, immune-mediated thrombocytopenia, inflammatory bowel disease, and Systemic Lupus Erythematosus [2,5,6,7]. These disorders result from the inability of immune cells to distinguish self from non-self, causing chronic inflammation, tissue damage, and impaired organ function, which significantly impacts the health and well-being of companion animals [3].

DC-SIGN (dendritic cell-specific intercellular adhesion molecule-3-grabbing non-integrin), a C-type lectin receptor expressed on dendritic cells and certain macrophages, plays a pivotal role in immune modulation and immune tolerance. Initially identified for its involvement in DC-T cell interactions via ICAM-3 binding [8], DC-SIGN has since been recognized as a key receptor that facilitates pathogen recognition, including viruses (e.g., HIV-1 [9], Feline Coronavirus [10,11], bacteria (e.g., *Mycobacterium tuberculosis* [12]), and fungi (e.g., *Candida albicans*) [13]. DC-SIGN mediates antigen uptake and presentation, bridging innate and adaptive immunity by internalizing pathogens and promoting antigen presentation to T cells in lymph nodes [14,15,16,17]. Its role extends to the regulation of immune responses, as it modulates cytokine environments and promotes immune tolerance, primarily through the induction of regulatory T cells (Tregs) via IL-10 production [18]. This ability to influence immune tolerance is particularly relevant in transplantation contexts, where DC-SIGN-expressing macrophages contribute to graft tolerance by inhibiting CD8+ T cell responses and expanding Tregs [18]. Furthermore, DC-SIGN is expressed in various human tissues, including dermal dendritic cells in the skin, T cell areas of lymph nodes, tonsils, spleen, DC-like cells in mucosal tissues such as the rectum, cervix, and uterus, specialized macrophages in the placenta and lung, and in allergic nasal polyps [8,19]. Therefore, utilizing DC-SIGN-mediated IL-10 secretion as a therapeutic approach has been explored in autoimmune diseases, allergic conditions, and transplant-related therapies [20,21,22]. Although the research on DC-SIGN’s roles in animals is limited, studies suggest that DC-SIGN homologs in various animal species also contribute significantly to immune modulation [23,24]. This highlights the potential of utilizing DC-SIGN-mediated IL-10 secretion in therapeutic approaches, not only in humans but also in veterinary medicine.

There is a need for effective and safe treatments for autoimmune and inflammatory diseases in companion animals [25]. The development of next-generation anti-inflammatory drugs should focus on restoring immune balance and controlling autoimmune responses while preventing excessive inflammation [26]. These drugs could provide a more refined mechanism of immune regulation compared to current treatments and play an essential role in improving the quality of life for companion animals. Recently, novel biomolecular-based therapies are being explored, which could enable more precise immune regulation [27]. These next-generation treatments may offer the potential to correct immune imbalances and prevent or suppress the onset of autoimmune diseases in companion animals [27,28].

This review will discuss the development of anti-inflammatory agents that harness the DC-SIGN-mediated IL-10 secretion effects within the innate immune system to promote immune tolerance. By modulating DC-SIGN, a key receptor on DCs and macrophages, it is possible to regulate immune responses and reduce inflammation. The review will focus on the possible application of these mechanisms in treating autoimmune and inflammatory diseases in companion animals and highlight challenges and future directions for DC-SIGN-targeted therapies.

## 2. Anti-Inflammatory Effects by DC-SIGN Mediated IL-10 Secretion

### 2.1. DC-SIGN Is a Marker of M2 Macrophages

Macrophages exhibit functional plasticity, dynamically polarizing into pro-inflammatory M1 and anti-inflammatory M2 phenotypes in response to environmental cues [29,30]. While M1 macrophages, activated by IFN-γ and LPS, are involved in pathogen defense and inflammatory responses, prolonged M1 activation can contribute to chronic inflammatory diseases [31,32]. In contrast, M2 macrophages, induced by IL-4 and IL-13, play a key role in resolving inflammation, promoting tissue repair, and maintaining immune homeostasis [33,34]. Their immunosuppressive properties, mediated by cytokines like IL-10 and TGF-β, are essential for controlling excessive immune activation and preventing autoimmune reactions [29,35].

DC-SIGN (dendritic cell-specific intercellular adhesion molecule-3-grabbing non-integrin), a C-type lectin receptor, has emerged as a crucial marker of M2 macrophages. While DC-SIGN is broadly expressed in dendritic cells, macrophages, and monocytes, its upregulation in M2 macrophages is linked to their immunomodulatory functions [36]. By engaging with ligands such as pathogen-associated molecular patterns (PAMPs) and host-derived molecules, DC-SIGN signaling promotes the production of anti-inflammatory cytokines, particularly IL-10, reinforcing M2 macrophage-mediated immune suppression [18,28,37,38].

Beyond its role in immune regulation, DC-SIGN is implicated in pathogen immune evasion and tumor progression. Certain pathogens, including HIV-1, exploit DC-SIGN to enhance viral transmission and modulate host immune responses [39]. In the tumor microenvironment, DC-SIGN-expressing M2 macrophages contribute to immune suppression, facilitating tumor escape from immune surveillance [21,40]. Additionally, in autoimmune diseases like rheumatoid arthritis, DC-SIGN influences macrophage-mediated inflammation, further highlighting its significance in immune homeostasis and disease progression [41].

Understanding the molecular mechanisms underlying DC-SIGN’s function in M2 macrophage polarization provides potential therapeutic opportunities to modulate immune responses in infectious, inflammatory, and cancer-related conditions.

### 2.2. DC-SIGN Mediated Anti-Inflammatory Effects by IL-10

**Figure 1:**
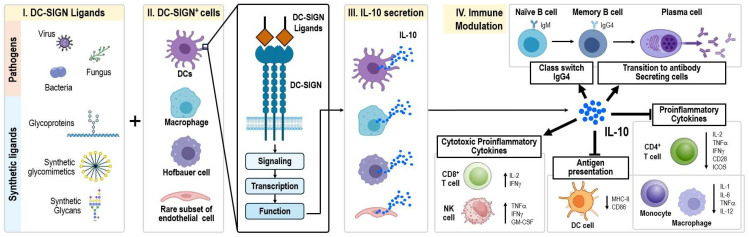
Anti-inflammatory therapeutic applications of DC-SIGN-mediated IL-10 secretion in human and potentially in veterinary medicine. DC-SIGN ligands include pathogens (viruses, bacteria, and fungi), glycoproteins on pathogen surfaces, and synthetic ligands like glycomimetics designed to mimic natural ligands. DC-SIGN is expressed on various cells, including dendritic cells, macrophages, Hofbauer cells, and some endothelial cells. When DC-SIGN binds to its ligands, it triggers signaling pathways that result in IL-10 secretion, an anti-inflammatory cytokine. IL-10 modulates immune responses by inhibiting pro-inflammatory cytokines, downregulating antigen presentation, and promoting IgG4 class switching in B cells. While IL-10 can enhance cytotoxicity in CD8+ T cells and NK cells, prolonged exposure may lead to T cell exhaustion, reducing immune response efficiency.

DC-SIGN, a C-type lectin receptor, plays a critical role in immune regulation by promoting IL-10 production upon engagement with ligands such as pathogen-associated molecular patterns (PAMPs) or host-derived molecules [28,42] (Figure 1). IL-10, a potent anti-inflammatory cytokine, inhibits pro-inflammatory pathways involving TNF-α and IL-12, thereby limiting excessive immune activation and preventing tissue damage during infections and autoimmune reactions [43,44,45].

IL-10’s anti-inflammatory role is evident in conditions such as sepsis, inflammatory bowel disease (IBD), and chronic infections. In sepsis, IL-10 reduces systemic inflammation and improves survival by suppressing pro-inflammatory cytokines like TNF-α and IL-6 [43,46,47]. In IBD, IL-10-deficient mice develop spontaneous colitis, demonstrating its critical role in maintaining gut immune homeostasis, while defective IL-10 receptor signaling increases susceptibility to the disease [48,49,50,51,52]. During chronic infections, such as HIV and hepatitis C, DC-SIGN-expressing dendritic cells and macrophages secrete IL-10 to limit tissue damage caused by persistent inflammation [53]. Similarly, in chronic inflammatory conditions like rheumatoid arthritis and COPD, IL-10 helps regulate immune responses and promote tissue repair [28,43,54].

Beyond these conditions, therapeutic applications of DC-SIGN and IL-10 are being explored. Recombinant IL-10 administration has shown promise in reducing disease severity in experimental autoimmune models by promoting immune tolerance [55]. Additionally, modulating DC-SIGN signaling and IL-10 production may help reshape the tumor microenvironment, potentially inhibiting tumor progression and metastasis [50,54,56,57].

However, while IL-10 prevents excessive inflammation, its dysregulation can impair immune responses, increasing susceptibility to infections [50,54,56,57]. The balance between resolving inflammation and maintaining immune defense is crucial, as disruptions in this regulation can lead to autoimmune diseases, chronic inflammatory disorders, or weakened pathogen resistance.

### 2.3. DC-SIGN^+^ Cells Are a Reservoir of IL-10

DC-SIGN^+^ cells appear to act as a reservoir of IL-10, rapidly responding to its stimulus (Figure 1) [18,21,37,58,59,60,61]. DC-SIGN^+^ cells are adept at producing IL-10 in response to various stimuli, including specific pathogens and their components, such as *Mycobacterium leprae* [37]. Upon antigen capture and internalization, these cells can undergo signaling cascades that activate transcription factors responsible for IL-10 production, such as NF-κB. The presence of certain ligands bound to DC-SIGN can enhance this signaling pathway, promoting higher levels of IL-10 secretion from the cells [58,62]. For example, studies have shown that mannose-capped lipoarabinomannan (ManLAM), a component of *Mycobacterium tuberculosis*, can bind to DC-SIGN and promote IL-10 production through the activation of the Raf-1-dependent signaling pathway [63].

The production of IL-10 by DC-SIGN^+^ cells is critical for shaping the immune environment. By releasing IL-10, these cells can exert profound effects on neighboring immune cells, dampening pro-inflammatory responses and promoting tolerance, which is particularly important in preventing excessive tissue damage during immune responses [64,65]. This regulatory function is particularly important in preventing excessive tissue damage during immune responses, as IL-10 helps to maintain a balance between pathogen clearance and tissue preservation [64,65]. The IL-10 produced by DC-SIGN^+^ cells can influence various immune processes, such as the differentiation and function of T cells. Specifically, IL-10 promotes the generation of regulatory T cells (Tregs) that play a key role in maintaining immune homeostasis and tolerance [62]. Additionally, the presence of IL-10 contributes to reducing the activation of effector T cells, thus preventing pathologic inflammation [62]. This dual role of IL-10 in promoting Treg differentiation and suppressing effector T cell activation highlights the importance of DC-SIGN^+^ cells in immune regulation [66]. The ability of DC-SIGN+ cells to produce IL-10 underscores their importance in both preventing autoimmunity and supporting appropriate immune responses against pathogens [44,67]. For instance, in the context of chronic infections, such as those caused by *Mycobacterium tuberculosis* or Leishmania, DC-SIGN^+^ cells can modulate the immune response to prevent excessive inflammation while still promoting pathogen clearance [68,69].

The regulatory function of IL-10 from DC-SIGN^+^ cells is particularly relevant in situations where inflammation needs to be carefully controlled, such as during infections or in autoimmune diseases. For example, in autoimmune conditions like rheumatoid arthritis or multiple sclerosis, the dysregulation of IL-10 production by DC-SIGN^+^ cells can contribute to disease progression [70,71]. Conversely, enhancing IL-10 production by these cells could offer therapeutic benefits in controlling inflammation and restoring immune balance [44,67].

Given their central role in immune regulation, DC-SIGN and its associated signaling pathways represent potential therapeutic targets for modulating immune responses in chronic inflammatory and autoimmune diseases. This is relevant not only in human medicine but also in veterinary medicine, where similar mechanisms of immune regulation are observed [72]. For example, targeting DC-SIGN to enhance IL-10 production could be a strategy to treat chronic inflammatory conditions in animals, such as inflammatory bowel disease or autoimmune skin disorders [50,62,73]. Therefore, DC-SIGN^+^ cells serve as a critical reservoir of IL-10, playing a key role in modulating immune responses and maintaining immune homeostasis. Their ability to produce IL-10 in response to various stimuli makes them essential for controlling inflammation and promoting tolerance, highlighting their potential as therapeutic targets in both human and veterinary medicine [44,67].

## 3. Implications of Utilizing DC-SIGN Mediated IL-10 Secretion in Immuno-Therapeutic Approach

Given its role in immune modulation, DC-SIGN has emerged as a potential target for immunotherapeutic strategies. DC-SIGN^+^ cells are a pivotal reservoir of IL-10, actively contributing to the modulation of immune responses through their production of this anti-inflammatory cytokine [37]. By serving as a source of IL-10, these cells play a central role in regulating and maintaining immune balance, making them crucial players in the immune system’s response to pathogens and in the prevention of immune-mediated tissue damage [43]. Also, it was reported that the activation of DC-SIGN can affect the transition from the inflammatory to the anti-inflammatory phenotype of DCs and alter the Th1/Th2 ratio [18,74,75,76]. The relative specificity of DC-SIGN for DCs and its dynamic immunoregulatory roles endow DC-SIGN with the potential as a molecular target to regulate the phenotype of the immune response [77]. In consequence, utilizing DC-SIGN-mediated IL-10 secretion in a therapeutic approach may be a feasible approach to induce immune tolerance in vivo [78]. Understanding their functions and interactions may provide insights into novel therapeutic approaches for various immune-related conditions.

### 3.1. Therapeutic Enhancement of IL-10 Production

Understanding the role of DC-SIGN^+^ cells as an IL-10 reservoir opens avenues for potential therapeutic interventions. For example, strategies aimed at enhancing IL-10 production from these cells could be beneficial in treating inflammatory and autoimmune disorders by promoting a more tolerogenic immune environment. For instance, tolerogenic DCs (tolDCs) have been recognized as vital for maintaining immune tolerance due to their capability to produce IL-10 and TGF-β [18]. Additionally, excretory-secretory products from the parasitic nematode Trichinella spiralis have been shown to induce tolDCs in vitro, which subsequently leads to increased IL-10 production. This suggests potential pathways for therapeutic interventions that could utilize DC-SIGN^+^ cells to foster a more tolerogenic immune response [18]. Therefore, exploiting the tolerogenic properties of DC-SIGN could lead to novel treatments for autoimmune conditions, allowing for more precise modulation of immune responses through the use of tolDCs.

### 3.2. Manipulation of DC-SIGN^+^ Cells in Tumor Immunity

Manipulating DC-SIGN^+^ cells may aid in enhancing anti-tumor immunity or responses against chronic infections by reducing their IL-10 output to promote more robust effector T cell responses [18]. Manipulating the functionality of DC-SIGN^+^ cells to decrease IL-10 production could enhance anti-tumor immunity [18]. Research indicates that vaccine-induced IL-10-producing DCs can impair the priming of anti-tumor responses. By managing the IL-10 output from these cells, it may be possible to promote more vigorous effector T cell responses necessary for combating tumors and chronic infections [79,80]. Also, DC-based vaccines that utilize DC-SIGN for targeted antigen delivery can enhance adaptive immune responses against cancer and infectious diseases [81].

### 3.3. Development of DC-SIGN Ligands (Table 1)

Developing ligands that target DC-SIGN has attracted considerable attention, particularly in the treatment of viral infections, cancer therapy, and vaccine development [82]. These ligands play a crucial role in strategically modulating immune responses, enhancing vaccine efficacy, and creating innovative therapeutic agents for combating specific pathogens [83]. Current research on DC-SIGN ligands focuses on multiple approaches, with a significant emphasis on glycomimetic ligands [84]. These synthetic carbohydrate-based ligands are designed to interact specifically with the Carbohydrate Recognition Domain (CRD) of DC-SIGN, enabling targeted delivery to DCs and modulation of the host immune response [85]. Studies have demonstrated that the multivalent presentation of glycomimetic ligands enhances binding to DC-SIGN and promotes clustering of specific receptors on the cell membrane, activating beneficial signal transduction pathways. Furthermore, the identification of pentamannoside epitopes that bind to DC-SIGN is under investigation, offering the potential to further refine immunomodulatory targeting strategies [86].

DC-SIGN ligands hold significant promise for preventing and treating diseases such as HIV [87]. Advances in their development could lead to robust immunotherapeutic strategies that enhance immune responses, overcome immune evasion mechanisms, and improve vaccine designs, ultimately providing more effective protection against a range of infectious diseases [83]. The type, specific ligands, source/pathogen, function, and corresponding references for the currently developed DC-SIGN ligands are summarized in Table 1. Each ligand plays a significant role in modulating the immune response through specific interactions with the DC-SIGN receptor.

**Table 1:** The type, specific ligands, source/pathogen, function, and corresponding references for the currently developed DC-SIGN ligands.

Ligand Type	Specific Ligands	Source/Pathogen	Function	References
Viral Ligands	HIV-1 gp120 (Human Immunodeficiency Virus type 1 Glycoprotein 120)	HIV	Attacks the body’s immune system	[88]
	KSHV(HHV-8)(Kaposi’s Sarcoma-associated Herpesvirus (Human Herpesvirus-8)	Oncovirus	Attacks the body’s immune system	[89]
	Ebola glycoprotein	Ebola virus	Causes severe inflammation and tissue damage throughout the body	[90]
	Dengue virus E protein	Dengue virus	Hijacks the host cell’s machinery	[91]
	Hepatitis C	Hepatitis C virus	Attacks the body’s immune system	[92]
	Measles	Measles virus	Infects the respiratory tract and then spreads throughout the body	[93]
	Herpes simplex	Herpes simplex virus	Painful blisters or ulcers that can recur over time	[94]
	Influenza A	Influenza A virus	Enable antigenic drift and antigenic shift	[95]
	SARS-CoV (Severe acute respiratory syndrome-related coronavirus)	SARS	In the formation and maintenance of the ribonucleoprotein complex	[96]
	Cytomegalo virus	Body fluids of someone else who’s infected	Expresses genes in a temporally controlled manner	[92,97]
	Japanese encephalitis virus	Mosquitoes	Inflammation of the active tissues of the brain caused by an infection or an autoimmune response	[98]
	West-Nile virus	Mosquitoes	Attacks the body’s immune system	[99]
	Phlebovirus	Naples phlebovirus	Attacks the body’s immune system	[100]
Bacterial Ligands	Lipopolysaccharides (LPS)	*Mycobacterium tuberculosis*	Establishes a permeability barrier that protects the cell from the entry of toxic molecules such as antibiotics and bile salts	[101]
	Mannose-capped lipoarabinomannan (ManLAM)	*Klebsiella pneumoniae*	Potential virulence factor which can bind to leukocytes and modulate immune responses	[39]
	Mycobacteria strains	Mycobacterium	Adapt its metabolism to the host environment and regulate entry into and exit from the cell cycle	[102]
	*Helicobacter pylori*	Transmitted person-to-person by saliva	Normalize stomach acid secretion, and regulate appetite	[74]
	*Mycobacterium bovis*	TB disease	Adapt its metabolism to the host environment and regulate entry into and exit from the cell cycle	[103]
Fungal Ligands	β-Glucans	*Candida albicans*	Stimulate the growth and activity of the desired natural intestinal microbiota, while inhibiting the growth of pathogens	[104]
	Mannans	*Aspergillus fumigatus*	Storage polysaccharides that provide energy for the growing seedling	[105]
	*Schistosoma mansoni*	Rodents and mammals	Infect the hosts by directly penetrating the skin	[106]
Glycans	High mannose glycans (Man9, Man5)	HIV, *Mycobacterium tuberculosis*	DC-SIGN binding to glycan ligands	[16,17]
	Neoglycoconjugate with multivalent Galβ1-4(Fucα1-3) GlcNAc (Le^x^) trisaccharides	Laboratory synthesis	Binding of recombinant DC-SIGN-Fc to CHO-glycosylation mutants	[107]
	Man9GlcNAc2-DPPE oligosaccharide neoglycolipid	Laboratory synthesis	DC-SIGN binding to ligands embedded in gel- and fluid-phase membranes	[36]
Glycoproteins	ICAM3 (Intercellular adhesion molecule 3)	Human cells	Found on the surface of leukocytes and constitutively expressed in these cells	[108]
	STD-NMR	NMR	Technique used to study protein-ligand interactions	[109]
	MGL (Macrophage galactose-type lectin)	Colorectal cancer	Facilitating the maintenance of immune homeostasis	[110]
	PSGL-1 (P-selectin glycoprotein ligand-1)	P-selectin	Plays a vital role in the immune system and in regulating white blood cell trafficking	[111]
	C1q	serum	Modulate the functions of immune and non-immune cells including dendritic cells and microglia	[112]
Synthetic Ligands	Mannose-based dendrimers, Synthetic glycomimetics	Laboratory synthesis	DC-SIGN binding to Mannose-based dendrimers, Synthetic glycomimetics	[113]

## 4. Development of Veterinary Anti-Inflammatory Agents Utilizing the DC-SIGN Mediated IL-10 Secretion

### 4.1. Overview of DC-SIGN Homologues in Animals

**Figure 2:**
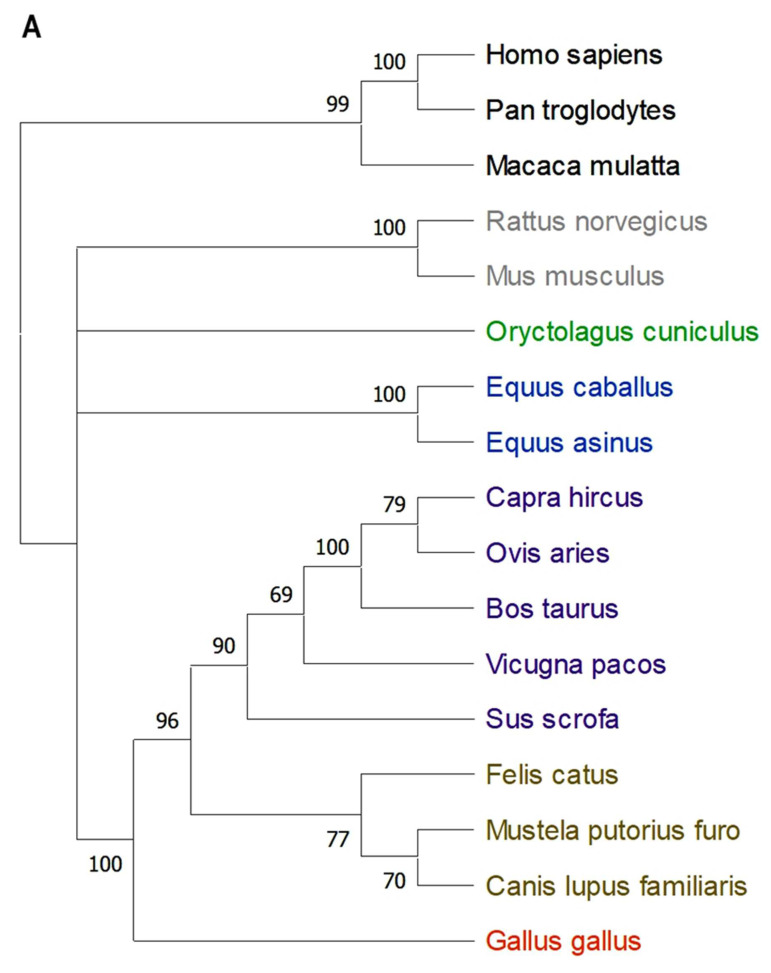

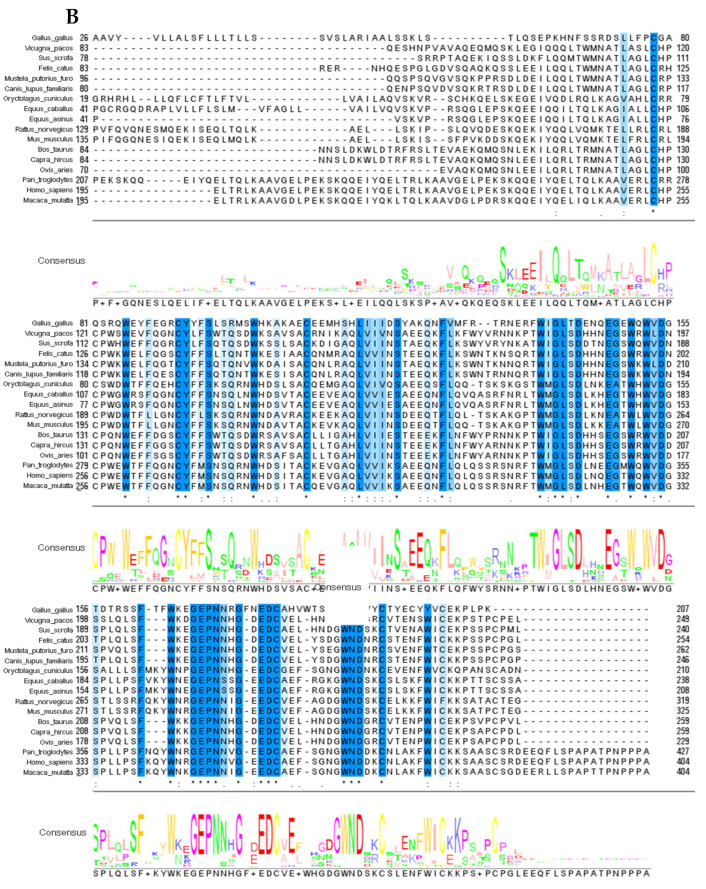
Overview of DC-SIGN Homologues in Animals. Comparison of the DC-SIGN/CD209 family in animals. (**A**) The phylogenetic tree illustrates the relationships of the CD209 family across various species. This tree was constructed using the maximum likelihood method based on the amino acid alignment of protein sequences. Bootstrap values were calculated from 100 repetitions. (**B**) The alignment of amino acid sequences of CD209 homologs was generated using Clustal Omega Multiple Sequence Alignment (EMBL-EBI website version) and visualized using Jalview (version 2.11.4.1). Consensus annotation is colored according to the Zappo format, which categorizes colors based on their physicochemical properties. All colored residues are conserved at a minimum of 50%. Darker-colored residues indicate a higher consensus sequence. An asterisk (*) signifies positions with fully conserved residues. A colon (:) denotes conservation among amino acid groups with similar properties, while a period (.) indicates conservation among groups with weakly similar properties. The NCBI accession number used for alignment and phylogenetic tree construction is as follows: human CD209 (NP_066978), chimpanzee CD209 (NP_001009064), rhesus monkey CD209 (NP_001028042), Norway rat-CD209b (NP_001163868), mouse CD209b (NP_081248), rabbit CD209 (XP_051691470), horse CD209L2 (XP_005611561), donkey CD209L2 (XP_014717460), goat CD209 (XP_005682468), sheep CD209L2 (XP_027825527), cow CD209 (NP_001139228), alpaca CD209 (XP_015096456), pig CD209 (NP_001123444), cat CD209 (XP_003981851), ferret CD209L2 (XP_044940870), dog CD209 (NP_001124304), and chicken hepatic lectin (NP_990815).

Various animal species possess homologs of DC-SIGN (CD209), which share structural and functional similarities in pathogen recognition and immune modulation [10,12,24,114,115,116,117,118,119]. Phylogenetic analysis reveals evolutionary relationships among species based on CD209 protein sequences (Figure 2A). Primates such as humans (*Homo sapiens*), chimpanzees (*Pan troglodytes*), and rhesus macaques (*Macaca mulatta*) cluster closely, reflecting their shared ancestry and complex immune systems. Rodents (*Rattus norvegicus*, *Mus musculus*) are widely studied for their immunological similarities to humans. Domesticated mammals, including cattle (*Bos taurus*), pigs (*Sus scrofa*), and horses (*Equus caballus*), show distinct evolutionary groupings, while carnivores such as dogs (*Canis lupus* familiaris) and cats (*Felis catus*) form a separate cluster. Birds, represented by chickens (*Gallus gallus*), occupy a distinct evolutionary branch, emphasizing divergence from mammals. Confidence scores support the reliability of these relationships, highlighting conserved and species-specific aspects of DC-SIGN evolution.

Multiple sequence alignments of CD209 across species (Figure 2B) reveal highly conserved and variable regions, crucial for understanding functional adaptations [120,121]. Conserved regions, indicated by blue shading, suggest essential structural and functional elements, while variable regions indicate species-specific adaptations. Sequence logos further illustrate conservation patterns across amino acid positions, aiding comparative immunology research.

DC-SIGN homologs belong to the C-type lectin receptor family, with species-specific variations influencing immune function. The mouse DC-SIGN family consists of nine members, including SIGN-R1 and SIGN-R3, which provide insights into human DC-SIGN functions [122,123]. SIGN-R1 recognizes sialylated glycans and activates the complement pathway [124], revealing human DC-SIGN’s role in immune homeostasis and tolerance [71,125,126]. SIGN-R3’s broader glycan-binding specificity suggests a diverse ligand repertoire for human DC-SIGN [127]. Differential expression patterns in mice have clarified tissue-specific DC-SIGN roles in humans, particularly in lymph nodes and mucosal surfaces [128,129].

Despite these advances, knowledge of DC-SIGN function in veterinary species remains limited. Differences in ligand specificity, signaling pathways, and immune functions among species underscore the need for species-specific studies to fully understand DC-SIGN’s role in veterinary medicine [130].

### 4.2. Anti-Inflammatory Effects Utilizing DC-SIGN Mediated IL-10 Secretion in Animals

Currently, there is limited research specifically focusing on anti-inflammatory therapies utilizing DC-SIGN-mediated IL-10 secretion in various animals. However, related studies provide insights that could inform future research in this area. Here, we introduce the representative DC-SIGN mediated anti-inflammatory effects in veterinary medicine as below.

#### 4.2.1. Pulmonary Fibrosis in Mice

Serum amyloid P activates DC-SIGN on mouse lung epithelial cells to produce IL-10, highlighting its distinct regulatory role in the innate immune system compared to C-reactive protein. This suggests that DC-SIGN could be a promising target for antifibrotic therapies against pulmonary fibrosis and acute lung injury in mice [88].

#### 4.2.2. Diastolic Dysfunction in Mice

Injection of the DC-SIGN ligand 1, an anti-inflammatory agent, prevented the progression of diastolic dysfunction over time in females but not in males of aged C57BL/6J mice [131].

#### 4.2.3. Atopic Dermatitis in Humans and Canine

DC-SIGN captures non-oxidized mannan-conjugated polymerized grass pollen allergens, promoting regulatory T cell induction via PD-L1 signaling, increasing IL-10/IL-5 ratios, and demonstrating efficacy and hypoallergenicity in both human and canine atopic dermatitis treatments [132].

#### 4.2.4. Melanoma in Canine

Canine DC-SIGN-targeted Ad5 vectors were developed in order to evaluate and compare the effectiveness of CD40- and DC-SIGN-targeted Ad5 vaccines against canine melanoma, a spontaneous and aggressive tumor which provides a stringent study system for the evaluation of antitumor immunotherapies [133].

#### 4.2.5. Application of DC-SIGN^+^ DCs in Feline

Feline DC-SIGN^+^ DCs, cultured from feline PBMCs without exposure to foreign proteins, create a solid foundation for developing in vivo vaccines and immunotherapies to treat cats infected with feline immunodeficiency virus (FIV) [134].

#### 4.2.6. Dry Eye Disease in Rabbit

Rabbit DC-SIGN may be responsible for the anti-inflammatory effect caused by IVIg or α-2,6 sialylated IVIg treatment, promoting clinical improvements in a rabbit dry eye model by regulating inflammatory cytokines [24].

Because DC-SIGN homologs are found on most animals (Figure 2), the application of DC-SIGN’s anti-inflammatory effects in veterinary medicine holds promise for improving the management of inflammatory and autoimmune diseases in animals. Utilizing DC-SIGN’s regulatory role in the immune system could lead to novel, targeted therapies that could offer new treatment options for managing inflammation in animals. In particular, glycoconjugates and receptor-specific antibodies targeting DC-SIGN-mediated IL-10 secretion offer distinct advantages to developing immune modulators or tumor vaccines. Glycoconjugates could reduce side effects through organic chemistry-based design and receptor-specific antibodies are effective at inducing immune responses in DC-targeted vaccination strategies [135,136]. Future research into DC-SIGN homologs and their functions across species will be critical for translating these findings into clinical practice.

## 5. Autoimmune and Immune-Mediated Diseases in Animals

In veterinary medicine, autoimmune and immune-mediated diseases affect various species [137]. Nearly 100 different autoimmune diseases have been reported in domestic animals, including dogs, cats, horses, and livestock (Table 2). These conditions often involve the immune system attacking the body’s own cells, leading to chronic inflammation and tissue damage [138], and can affect various systems in the body, such as the skin, blood, joints, and even the nervous system [139]. Ordinarily, the immune system remains tolerant of self-antigens through a number of mechanisms, beginning with the deletion of autoreactive T cells in the thymus and B cells in bone marrow [140]. However, autoimmune diseases are caused by the development of inappropriate immune responses directed against host antigens [141]. Autoimmune diseases are often referred to as ‘immune-mediated’ diseases in veterinary medicine [142,143]. Since human DC-SIGN is distributed throughout systemic tissues [144], it is likely that animal DC-SIGN is also distributed systemically in animals. Therefore, administering animal DC-SIGN ligands in cases of inflammatory diseases in animals could stimulate DC-SIGN, leading to the production of IL-10. This IL-10 production may exert immunosuppressive or immunomodulatory effects, potentially resulting in the treatment of these diseases [54,145]. Here, we introduce the representative autoimmune or immune-mediated diseases in companion animals that could potentially be treated through DC-SIGN-mediated IL-10 secretion as below.

**Table 2:** Representative autoimmune and immune-mediated diseases in companion animals.

No.	Disease	Description	Current Treatment	Challenges	References
1	Immune-mediated Hemolytic Anemia (IMHA)	Immune destruction of erythrocytes due to self-antigens.	Glucocorticoids, cyclosporine, azathioprine, mycophenolate mofetil, danazol, IVIG.	Adverse effects: hepatotoxicity, myelosuppression, hypercoagulability, UTIs.	[146,147]
2	Immune Thrombocytopenia (ITP)	Premature destruction of platelets by antibody-mediated phagocytosis.	Glucocorticoids, vincristine.	Severe bleeding complications before treatment becomes effective.	[148,149,150]
3	Evan’s Syndrome	Concurrent IMHA and ITP.	Glucocorticoids, IVIG, leflunomide.	Risks of glucocorticoids: diabetes, gastrointestinal hemorrhage; IVIG: anaphylactic shock; leflunomide: myelosuppression.	[147,151]
4	Toxic Epidermal Necrolysis (TEN)	Life-threatening mucocutaneous disease linked to drug reactions.	Drug withdrawal, immunosuppressants, IVIG.	Offending drugs may not be identifiable; IVIG: immune-mediated anaphylaxis; immunosuppressants: myelosuppression.	[147,152]
5	Erythema Multiforme (EM)	Acute mucous membrane and skin reaction.	Drug discontinuation, glucocorticoids, immunosuppressants.	Lack of controlled studies on therapeutic drug efficacy.	[153]
6	Stevens–Johnson Syndrome (SJS)	Rare immune-mediated skin disorder with widespread mucocutaneous lesions.	Glucocorticoids, supportive treatment, IVIG.	Limited evidence for treatment efficacy; potential harm from therapies.	[154,155].
7	Acquired Myasthenia Gravis (MG)	Neuromuscular disease caused by autoantibodies targeting acetylcholine receptors.	Anticholinesterase drugs, glucocorticoids, IVIG, supportive care.	High mortality from complications before remission.	[156,157]
8	Immune-mediated Polyarthritis (IMPA)	Non-infectious inflammatory polyarthropathy.	Glucocorticoids, immunomodulators.	Glucocorticoids cause collagen catabolism, worsening arthropathy.	[158]
9	Pemphigus Complex	Rare mucocutaneous autoimmune diseases causing flaccid blisters and erosions.	Glucocorticoids, IVIG, immunosuppressants, gold therapy.	Limited response to glucocorticoids; minimal improvement with additional therapies.	[159,160]
10	Lupus Erythematosus (LE)	Chronic autoimmune disease affecting multiple systems.	Immunosuppressants, glucocorticoids, dietary changes, antibiotics, topical treatments.	Limited efficacy of immunosuppressive therapies.	[161,162]
11	Inflammatory Bowel Disease (IBD)	Chronic gastrointestinal inflammation with unknown etiology.	Dietary changes, immunosuppressive drugs, antibiotics.	Immunosuppressants cause GI side effects; antibiotics disrupt gut microbiota.	[147,163]
12	Granulomatous Meningoencephalitis (GME)	Severe inflammatory disease of the CNS.	Corticosteroids, immunosuppressants.	High mortality within days of diagnosis despite aggressive treatment.	[164,165]
13	Keratoconjunctivitis Sicca (KCS)	Autoimmune-mediated damage to lacrimal glands, leading to dry eye disease.	Tear stimulants, glucocorticoids, cyclosporine.	Poor response to cyclosporine; long-term glucocorticoids risk corneal ulceration.	[166,167]
14	Sjögren-like Syndrome	Autoimmune inflammation of exocrine glands causing dry eye and mouth.	Tear substitutes, immunosuppressants, antibiotics.	Secondary bacterial infections; limited glandular recovery.	[168,169]
15	Sudden Acquired Retinal Degeneration Syndrome (SARDS)	Sudden vision loss due to photoreceptor dysfunction with neuroendocrine and autoimmune factors.	Symptom management, supportive care, treatments for secondary conditions.	Poor understanding of pathogenesis; limited treatment efficacy.	[166,170,171]

## 6. The Significance of Comparative Medicine and Translational Research Between Veterinary and Human Health

Current therapeutic approaches for autoimmune and immune-mediated diseases in veterinary medicine include corticosteroids [172,173,174,175], NSAIDs [176,177,178], mesenchymal stem cell therapy [25,179], monoclonal antibody therapies [180,181], gene therapy [182,183,184], cytokine therapy [185,186,187,188,189], and human intravenous immunoglobulin (hIVIG) [190,191]. While these treatments offer benefits, their limitations include severe side effects, high costs, variable efficacy, and the need for rigorous research to optimize protocols and expand applications [82,83,84,85,86,87,89,90,91,92,93,94,95,96,97,98,99,100,101,102,103,104,105,106,107,108,109,110,111,112,113,114,115,116,117,118,119,120,121,122,123,124,125,126,127,128,129,130,131,132,133,134,135,136,137,138,139,140,141,142,143,144,145,146,147,148,149,150]. This highlights the demand for safer, more effective, and tailored therapeutic strategies in veterinary practice. In particular, hIVIG holds potential as an adjunctive therapy for immune-mediated diseases in veterinary medicine, such as IMHA, ITP, and myasthenia gravis [150,157,192]. However, its use is hindered by high costs, limited availability, and insufficient research on dosing and outcomes [152,193,194,195]. Additionally, a shorter half-life in dogs and adverse effects, including hypersensitivity, hemolytic anemia, and rare complications like TEN and sepsis, present significant challenges [196,197,198]. Further research is necessary to establish safe and effective veterinary protocols [199,200] (Table 3).

**Table 3:** Recent veterinary studies of hIVIG application.

Conditions	Study Design	Dose	Outcomes	References
IMHA	Prospective (14 dogs)	1 g/kg daily, up to 2 days	No significant advantage was found	[196]
ITP	Prospective (18 dogs)	0.5 g/kg in 6% solution over 6 h; once	Significant reduction in platelet count recovery time, duration of hospitalization	[201]
ES	Case report of 1 dog	1.3 g/kg in a 5% solution over 8 h; once	Complete remission of ES; No relapse over 19-month follow-up	[151]
Cutaneous disease	Case report of 2 dogs	1 g/kg in over 4 h daily, for 2 days	Condition improved dramatically 4 days after administration	[152]
EM	Case report of 1 dog	0.45 mg/kg over 4.5 h once	Remarkable improvement post 48 h of administration	[202]
SJS	Case report of 1 dog	0.51 g/kg over 7 h; once	The lesions healed over 7 days and demeanor is also improved.	[154]
PF	Case report of 1 dog	0.5 g/kg over 5 h, for 4 days; following a total of 7 times infusion at the same dose after discharge	Markedly improved; remained asymptomatic 4.5 months after the final infusion.	[160]
MG	Case series of 3 dogs	0.5 g/kg over 6 h;	Responded initially, but clinical signs recurred	[197]

IMHA, immune-mediated hemolytic anemia; ITP, immune-mediated thrombocytopenia; ES, Evan’s syndrome; EM, erythema multiforme; SJS, Stevens–Johnson syndrome; PF, Pemphigus foliaceus; MG, Myasthenia gravis.

Rodent models have limitations due to anatomical and physiological differences from humans, which can hinder their relevance for diseases where symptoms and treatment responses do not closely align [203]. However, naturally occurring diseases in large animals are typically diagnosed based on their symptoms and often share similar molecular mechanisms with human diseases [204,205]. Studying diseases common to humans and animals through a comparative approach can overcome practical, ethical, and biological limitations in human and rodent research, offering valuable insights into pathological processes and significantly advancing clinical practice and treatment development in both veterinary and human medicine [206,207,208]. Many diseases, such as cancer, diabetes, cardiovascular diseases, and infectious diseases, manifest in both humans and animals [209].

Also, naturally occurring cancers in pet dogs and humans share similarities in histology, genetics, molecular targets, behavior, and therapy response. Dogs suffer from common human types of cancer, including lymphoma, melanoma, osteosarcoma, and prostate cancer, and have been extensively studied as a large animal model [210,211,212,213,214]. As canine patients are immunocompetent and outbred, research on canine cancers offers unique insights that complement human and rodent studies, contributing to gene identification, environmental risk assessment, tumor biology understanding, and novel therapeutic development [215]. Canine osteosarcoma shares pathological and genetic similarities with human osteosarcoma, making dogs valuable models for studying disease progression and testing potential therapies [215]. The canine melanoma model using DC-SIGN-targeted Ad5 vectors represents a crucial bridge between murine and human immunotherapy studies [216]. Monoclonal antibody therapies developed for canine lymphoma have informed similar treatments in human oncology [215].

In addition, success in DC therapy against these canine cancers promises not only advancement in cancer treatment in veterinary medicine but also provides effective information that advances DC therapy in humans. There are a few reports concerning the development of DCs in canine peripheral blood cell culture [217,218,219]. The study of feline immunodeficiency virus (FIV) offers insights into human immunodeficiency virus (HIV) due to shared mechanisms of immune evasion and pathogenesis [220]. Experiments using animal models of infection, such as those involving *Largemouth bass* and *Mycobacterium* spp., have revealed the significance of DC-SIGN in mediating immune responses and pathogen recognition [119]. Likewise, the application of One Health approaches emphasizes the interconnectedness of animal, human, and environmental health, facilitating cross-species solutions to antimicrobial resistance and zoonotic diseases [221,222]. All of these findings underscore the necessity of investigating DC-SIGN functionality across various species.

Veterinary research holds immense potential to benefit human medicine, yet this opportunity is often overlooked due to the predominant flow of knowledge and funding from human to veterinary medicine. Greater attention to veterinary advancements, particularly in studying naturally occurring animal diseases, could significantly enhance human medical research. This is exemplified by the contrast in protozoal vaccine development, where veterinary medicine has achieved multiple successes, while human medicine has none [223]. Moreover, veterinary insights often reveal innovative strategies for disease management, such as immune modulation and regenerative medicine, which are later adapted for human use [215]. In comparison with human diseases, vaccine development for animals has practical advantages such as the ability to perform experiments in the natural host, the option to manufacture some vaccines in vivo, and lower safety requirements [223]. Therefore, these insights emphasize the importance of understanding the anti-inflammatory effects of animal DC-SIGN in order to develop more effective treatments for inflammatory and autoimmune diseases that can be applied to both veterinary and human medicine. The treatment strategies for inflammatory and autoimmune diseases in dogs and humans predominantly depend on broad-spectrum immunosuppressive medications, which may cause significant side effects in certain patients [143,224]. This concern is especially notable in individuals and animals with autoimmune hemolytic anemia (AIHA), where prolonged and high doses of these drugs are often necessary [2]. The exploration of both animal and human DC-SIGN ligands offers promising pathways for the development of targeted immune suppressors, alleviating adverse drug effects. For instance, using rabbit DC-SIGN in combination with 2,6 IVIG has been shown to promote the generation of IL-10, demonstrating the potential for advancements in immune therapies through comparative research in veterinary and human medicine.

## 7. Conclusions and Future Perspectives

Finding a safe and effective drug to control inflammation has been a challenge and therefore, many animal models have been developed for the evaluation of drugs having anti-inflammatory properties [225]. The identification of DC-SIGN has significantly advanced our understanding of immune regulation and facilitated the development of therapeutic strategies that leverage its distinctive characteristics. DC-SIGN interacts with specific ligands to induce anti-inflammatory effects by promoting the production of cytokines such as IL-10 and TGF-β. These cytokines play a critical role in suppressing excessive inflammatory responses and facilitating immune tolerance. DC-SIGN homologs in animals share similar structures and immune functions, providing insights for cross-species applications. Therefore, the development of DC-SIGN ligands offers the advantage of mutual applicability between humans and animals, enabling the creation of immune suppressors for both. DC-SIGN ligands, such as glycomimetics and pentamannoside epitopes, are being developed to target DC-SIGN. These ligands enhance receptor binding, modulate immune responses, and hold promise for treating inflammatory and autoimmune diseases. Their development is crucial for advancing immunotherapies, offering innovative strategies to regulate immune activity and alleviate disease symptoms. The immunomodulatory properties of DC-SIGN hold substantial promises for enhancing disease management and promoting animal health in veterinary medicine. Furthermore, continued investigation into DC-SIGN homologs and their clinical applications may yield valuable insights that contribute to advancements in human medicine.
